# Inhibition of acyl-CoA binding protein (ACBP) by means of a GABA_A_Rγ2-derived peptide

**DOI:** 10.1038/s41419-024-06633-6

**Published:** 2024-04-06

**Authors:** Gerasimos Anagnostopoulos, Ester Saavedra, Flavia Lambertucci, Omar Motiño, Jordan Dimitrov, David Roiz-Valle, Victor Quesada, Karla Alvarez-Valadez, Hui Chen, Allan Sauvat, Yan Rong, Uxía Nogueira-Recalde, Sijing Li, Léa Montégut, Mojgan Djavaheri-Mergny, Maria Castedo, Carlos Lopez-Otin, Maria Chiara Maiuri, Isabelle Martins, Guido Kroemer

**Affiliations:** 1 Centre de Recherche des Cordeliers, Equipe labellisée par la Ligue contre le cancer, Université de Paris, Sorbonne Université, Inserm U1138, Institut Universitaire de France, Paris, France; 2 Metabolomics and Cell Biology Platforms, Institut Gustave Roussy, Villejuif, France; 3 Departamento de Bioquímica y Biología Molecular, Fisiología, Genética e Inmunología, Instituto Universitario de Investigaciones Biomédicas y Sanitarias (IUIBS), Universidad de Las Palmas de Gran Canaria, Las Palmas de Gran Canaria, Paris, Spain; 4 Centre de Recherche des Cordeliers, INSERM, CNRS, Sorbonne Université, Université Paris Cité, Paris, France; 5 Departamento de Bioquímica y Biología Molecular, Instituto Universitario de Oncología (IUOPA), Universidad de Oviedo, Oviedo, Spain; 6 Centro de Investigación Biomédica en Red de Cáncer (CIBERONC), Madrid, Spain; 7 Faculté de Médecine, Université de Paris Saclay, Kremlin Bicêtre, Paris, France; 8 Grupo de Investigación en Reumatología (GIR), Instituto de Investigación Biomédica de (INIBIC), Fundación Profesor Novoa Santos, A Coruña, Spain; 9 Facultad de Ciencias de la Vida y la Naturaleza, Universidad Nebrija, Madrid, Spain; 10 Department of Molecular Medicine and Medical Biotechnologies, University of Napoli Federico II, 80131 Naples, Italy; 11 Institut du Cancer Paris CARPEM, Department of Biology, Hôpital Européen Georges Pompidou, AP-HP, Paris, France

**Keywords:** Macroautophagy, Mechanisms of disease

## Abstract

Acyl-CoA binding protein (ACBP) encoded by *diazepam binding inhibitor* (DBI) is an extracellular inhibitor of autophagy acting on the gamma-aminobutyric acid A receptor (GABA_A_R) γ2 subunit (GABA_A_Rγ2). Here, we show that lipoanabolic diets cause an upregulation of GABA_A_Rγ2 protein in liver hepatocytes but not in other major organs. ACBP/DBI inhibition by systemically injected antibodies has been demonstrated to mediate anorexigenic and organ-protective, autophagy-dependent effects. Here, we set out to develop a new strategy for developing ACBP/DBI antagonists. For this, we built a molecular model of the interaction of ACBP/DBI with peptides derived from GABA_A_Rγ2. We then validated the interaction between recombinant and native ACBP/DBI protein and a GABA_A_Rγ2-derived eicosapeptide (but not its F77I mutant) by pull down experiments or surface plasmon resonance. The GABA_A_Rγ2-derived eicosapeptide inhibited the metabolic activation of hepatocytes by recombinant ACBP/DBI protein in vitro. Moreover, the GABA_A_Rγ2-derived eicosapeptide (but not its F77I-mutated control) blocked appetite stimulation by recombinant ACBP/DBI in vivo, induced autophagy in the liver, and protected mice against the hepatotoxin concanavalin A. We conclude that peptidomimetics disrupting the interaction between ACBP/DBI and GABA_A_Rγ2 might be used as ACBP/DBI antagonists. This strategy might lead to the future development of clinically relevant small molecules of the ACBP/DBI system.

## Introduction

Human acyl-coenzyme A binding protein (ACBP) is encoded by *diazepam binding inhibitor* (*DBI*) gene. This double name, ACBP/DBI, reflects the dual function of this small (~10 kDa) protein, which binds to medium- and long- chain acyl coenzyme A (CoA) esters within cells, but can also be secreted into the extracellular space. ACBP/DBI acts on diazepam receptors on the surface of cells in an auto-, para- or endocrine fashion [1–3]. It is phylogenetically conserved and appears to act as an extracellular inhibitor of autophagy, meaning that its neutralization enhances autophagy in yeast [4], plants [5, 6], nematodes [7], mice [8, 9] and human cell cultures [8].

ACBP/DBI expression is upregulated in metabolic human diseases including obesity [8, 10], diabetes [11] and non-alcoholic steatohepatitis [12]. Mouse experimentation has shown that the neutralization of ACBP/DBI has positive effects on metabolism, reducing appetite and blunting high-fat diet-induced obesity and diabetes [8]. ACBP/DBI inhibition also prevents liver damage from various insults including mechanical damage (ischemia/reperfusion, bile duct ligation), dietary stress (high-fat, Western-style or methionine/choline-deficient diet) and hepatotoxins (acetaminophen, carbon tetrachloride, concanavalin A) [9].

These metabolic and hepatoprotective effects have been obtained through two different strategies of ACBP/DBI inhibition. At the genetic level, ACBP/DBI can be safely knocked out in adult mice using a tamoxifen-inducible Cre recombinase that excises the floxed *Acbp/Dbi* exon 2 in all cells of the body [8]. Alternatively, *Gabrg2* can be safely mutated in one single amino acid (F77I) at a constitutive level to abolish its interaction with its ligand ACBP/DBI [13, 14]. At the immunological level, ACBP/DBI autoantibody productions can be induced by a specific autovaccination schedule that breaks self-tolerance, leading to the generation of neutralizing IgG anti-ACBP/DBI antibodies [15]. Alternatively, ACBP/DBI can be neutralized by the systemic (intraperitoneal) injection of specific poly- or monoclonal antibodies [13]. The fact that antibodies and receptor mutations are as efficient in neutralizing pathogenic functions of ACBP/DBI, as the knockout of *Acbp/Dbi* [9], supports the importance of extracellular (as opposed to intracellular) ACBP/DBI in mediating obesogenic, pro-diabetic and hepatotoxic effects. Indeed, a mutated ACBP/DBI protein that loses its capacity to interact with acyl-CoA (and thus the intracellular function of the protein) is as efficient as the unmutated ACBP/DBI in inducing appetite in mice [16].

Driven by this, we wondered whether it might be possible to design peptides that inhibit extracellular ACBP/DBI and hence act as ACBP/DBI antagonists. Logically, we considered the possibility of designing such peptides from the ACBP/DBI receptor GABA_A_R. Here, we employed this strategy to design a peptide that flanks F77 from GABA_A_Rγ2 and interacts with recombinant or natural ACBP/DBI to block the metabolic effects of ACBP/DBI in vitro (on primary hepatocytes) and in vivo (in mice), specifically in terms of appetite control and hepatotoxicity. These findings have important consequences for the future design of small molecules affecting the ACBP/DBI-GABA_A_R interaction.

## Materials and methods

### Mouse experiments

All mice used in this study were bred and housed in a pathogen-free, temperature-controlled environment with 12 h light/dark cycles following the FELASA guidelines, EU Directive 63/2010, and French legislation. C57BL/6 8–12-week-old male mice were acquired from Envigo (Envigo, Gannat, France) or Charles River (Charles River Laboratory, Lentilly, France). Tamoxifen-inducible whole-body knockout of floxed *Acbp/Dbi*^f/f^ (ACBP KO: *UBC-cre/ERT2*::*Acbp/Dbi*^fl/fl^; ACBP WT control: *Acbp/Dbi*^fl/fl^ without Cre [8] were bred in the CRC animal facility. Mice were housed in SPF conditions with a 12 h light/dark cycles, temperature-controlled environment and received water and food *ad libitum*. Mice received a regular chow (RCD, Safe, #A04), 60% high-fat (Safe, #260 HF), high-fat/high-sucrose/1.25% cholesterol Western (WD) (Teklad, #MD.120528), or methionine/choline-deficient (MCD) (Essingen, #AIN-76) diet. In one series of experiments, C57BL/6 transgenic mice expressing microtubule-associated proteins 1A/1B light chain 3B (hereafter referred to as LC3) fused to green fluorescent protein (GFP) [17] were kindly provided by Noboru Mizushima (National Institute for Basic Biology, Okazaki, Japan). Mice were randomized into experimental groups, though without blinding the investigators.

#### Food intake experiments

Prior to experimentation, mice were subjected to 24 h starvation followed by individual housing and acclimatization in individual cages (2 h). Subsequently, mice were intravenously (i.v.) injected with recombinant ACBP/DBI protein (RecACBP/DBI) (total volume of 200 μL, 0.5 mg/kg body weight), either alone or in combination with GABA_A_Rγ2 peptides (5× molar excess). Cumulative food intake was then analyzed as previously described [16]. Prior to i.v. injections, the RecACBP mixes were pre-incubated (16 h, 4 °C) with GABA_A_Rγ2 peptides.

#### Liver autophagy activation by GABA_A_Rγ2 peptides

Ten-week-old C57BL/6 male mice were injected with GABA_A_Rγ2 peptides (5 mg/kg, i.v., dissolved in PBS) 4 h and 30 min before sacrifice. For inhibition of the autophagy flux, leupeptin (Leu, 30 mg/kg B.W., dissolved in PBS) was injected 2 h before sacrifice via an intraperitoneal (i.p.) injection. Control animals were i.v. injected with the vehicle (DMSO, 0.01%) and i.p. injected with PBS. All animals were sacrificed, and livers were snap-frozen in liquid nitrogen and stored at −80 °C.

#### Concanavalin A-induced acute liver injury

GABA_A_Rγ2 peptides (5 mg/kg, i.v., dissolved in DMSO) were injected in 12-week-old C57BL/6 male mice 90 min before the injection of 12 mg/kg Concanavalin A (ConA, i.v., Sigma Aldrich, # C5275) [9].

#### Primary mouse hepatocyte isolation and culture

Hepatocytes were isolated from control *Acbp/Dbi f/f*, *Acbp/Dbi* KO and GFP-LC3-expressing transgenic mice by perfusion through the inferior vena cava with Hank’s Balanced Salt Solution (HBSS 1×) (Gibco, # 14025092), 1 mM HEPES (pH = 7.4), 0.2 mM EGTA, and William’s E medium (Sigma) containing 7.5 mg collagenase from *Clostridium histolyticum* (Type IV, 0.5–5.0 FALGPA units/mg solid, ≥125 CDU/mg solid, Sigma). After filtration through a 100 µm cell strainer and successive centrifugations at 30 × *g*, 4 °C for 5 min, cells were resuspended in the following attachment culture medium: DMEM/F12, 20 mM HEPES (pH = 7.4), 5 mM glucose, 10% FBS (Sigma), 5 mg/mL BSA, 100 U/mL penicillin, and 100 μg/mL streptomycin. The cells were then purified by density gradient centrifugation using an isotonic solution of Percoll (GE Healthcare Bio-Sciences AB, Uppsala, Sweden). Cell viability was assessed using Trypan blue exclusion. Cells were plated in a 96-well plate overnight to facilitate cell attachment before proceeding with experiments [18].

#### Hepatocyte autophagy activation by GABA_A_Rγ2 peptides

GFP-LC3-expressing hepatocytes were cultured at 37 °C in complete medium supplemented with either vehicle or GABA_A_Rγ2-derived peptides (10 µg/mL) for 18 h. Leupeptin (100 μM) was added 4 h before fixation. Cells were stained with Hoechst 33342 solution (Thermo Scientific™, #62249, 1 µg/mL) and fixed with 4% paraformaldehyde (PFA) solution. Both blue (Hoechst-derived) and green (GFP-LC3-derived) fluorescence were imaged using a Zeiss LSM 710 confocal microscope. Images were analyzed using R software.

### Liver histology

Mouse liver samples were fixed in 20–30 mL 4% v/v formaldehyde solution (4 °C) for 24–48 h, followed by dehydration (incubation in gradually increasing ethanol solutions; 70–100% v/v) and paraffin inclusion as previously described [14]. Five-micrometer sections were stained using hematoxylin and eosin (HE), or immunohistochemically using anti-mouse GABRG2 (Antibodies online #AA41–140) according to standard procedures. Sections were then scanned by means of a Zeiss Lame Axioscan (objective: ×20). Images were analyzed using Image J or Zen software.

### Immunofluorescence in liver sections

Analysis of murine GABA_A_Rγ2 levels was performed in livers via immunofluorescence. Livers were fixed in 20–30 mL 4% v/v formaldehyde solution (4 °C) for 24 h, followed by 30% sucrose treatment (4 °C) overnight. Samples were embedded in Tissue-Tek OCT compound (Sakura Finetechnical) and stored at −80 °C as previously described [9]. Five-micrometer-thick tissue sections were prepared with a cryostat (Leica Microsystems, #CM3050S), air-dried for 30 min, and washed three times in PBS for 5 min. Sections were then permeabilized (0.2% Triton X-100) for 10 min, washed three times in PBS for 5 min, and blocked (0.1% Triton X-100, 10% horse serum, 1% BSA, room temperature) for 2 h. Samples were finally incubated overnight in the primary antibody mix (Mouse Gabrg2: Abcam #ab87328, Mouse F4/80: MCA497G, Mouse Albumin: R&D Systems #AF3329). After washed in PBS, samples were incubated in secondary antibody mix (Anti-rabbit AF647 1:500, Anti-goat AF488 1:500) at room temperature for 1 h and mounted with DAPI Fluoromount-G® antifading medium (SouthernBiotech, AL, USA). The slides were scanned using a LSM 710 confocal fluorescence microscope (Carl Zeiss, Jena, Germany). Hepatic GABA_A_Rγ2 levels were quantified across all sections of liver tissue samples visualized in fields of view from four mice per group, utilizing R Software.

### Chemicals, cell lines, culture conditions

Media and cell culture supplements were purchased from Gibco-Invitrogen (Carlsbad, CA, USA). Plasticware was purchased from Corning B.V. Life Sciences (Schiphol-Rijk, The Netherlands). Unless reported otherwise, the cell line used in this study was cultured at standard conditions (37 °C, 5% CO_2_, Dulbecco’s modified Eagle’s medium containing 10% fetal bovine serum, 10 mM HEPES buffer, 100 mg/L sodium pyruvate, 100 U/mL penicillin G sodium, and 100 µg/mL streptomycin sulfate). 2-deoxy-D-glucose (# D8375) and rotenone (# R8875) were purchased from Sigma (Burlington, MA, USA). Human hepatocellular carcinoma (Huh-7) cell line was used for in vitro experiments. Cells were plated in 6- or 96-well plates and grown for 24 h before treatments.

#### Stable shAcbp-expressing Huh-7 cell line

shRNAs directed against Acyl-CoA Binding Protein (*Acbp*), *shAcbp-3* (TRCN0000105050), as well as a negative control shRNA, were inserted into the pLKO.1-puro lentiviral vector obtained from Sigma (Burlington, MA, USA). Approximately 2 × 10^5^ Huh-7 cells were seeded in a six-well plate with Dulbecco’s Modified Eagle’s Medium (DMEM) supplemented with 10% fetal bovine serum (FBS) until reaching 60–70% confluence. Subsequently, the cells were transduced with lenti-shRNA particles (25–35 μL) in 1 mL fresh medium (DMEM, 10% FBS, 5 μg/mL Polybrene, Sigma, Burlington, MA, USA, # TR-1003). Following transduction, the medium was replaced 24 to 48 h later (DMEM, 10% FBS), and cells were incubated for an additional day. Puromycin selection (10 µg/mL, Thermo Fisher Scientific, Carlsbad, CA, USA, #A1113803) was applied for 1 week to isolate the successfully transduced Huh-7 cells. For the isolation of monoclonal stable cell lines, single-cell sorting was conducted following standard protocols.

### WST-8 conversion

WST-8 conversion assays were conducted utilizing the Cell Counting Kit-8 (Sigma-Aldrich, #96992). Cells were seeded in a 96-well plate at a density of 3000 cells per well and incubated overnight for adherence. Then, the culture medium was removed, and fresh medium containing the specified treatments was added to the wells. Immediately following the addition of treatments, 10 μL of CCK-8 reagent was introduced to each well. Subsequently, the cells were incubated for 4.5 h at 37 °C to facilitate the conversion of WST-8 in response to cellular activity. Following the incubation period, the absorbance at 450 nm was quantified using a VICTOR® Nivo™ microplate reader. This measurement served as an indicator of WST-8 conversion, reflecting cellular metabolic activity.

### Immunoblot

Protein lysates were prepared from liver, epididymal white adipose tissue (eWAT), brown adipose tissue (BAT), muscle, heart or Hep55.1c cells. Immunoblot analysis was performed for GABA_A_Rγ2 (Abcam #ab87328), mouse ACBP/DBI (Abcam, #ab231910), Human ACBP (Santa-Cruz #sc-376853), MAP1LC3B (#2775, Cell Signaling Technologies), and β-actin (Abcam, #ab49900), as previously described. [14].

### Pull down experimentation assay

The physical interaction between the native ACBP and GABA_A_Rγ2 peptides was examined by standard immunoprecipitation (IP) and immunoblotting protocols. In detail, liver protein extracts (500 μg) were immunoprecipitated on protein A/G-Sepharose beads (Merck Millipore, Burlington, MA, USA, #GE17-0618-01) coated with (1× 2× 4×) either GABA_A_Rγ2 wild type (WT) or mutated (F77I) eicosapeptide (Biosynth ltd) or its negative control (PBS). Each IP reaction was incubated overnight (4 °C) in a rotation chamber followed by three consecutive rounds of PBS washing the next day. Each washing round included a PBS resuspension of the pellet and a re-centrifugation (12,000 × *g*, 4 °C). Finally, beads were resuspended in 20 μL of NUPAGE 4× buffer (Life Technologies, CA, USA, #NP0008), heated at 100 °C (10 min), followed by standard immunoblotting for the ACBP protein (Abcam #ab231910).

### Computational protein modeling

Peptide-protein docking structure between mouse ACBP/DBI and GABA_A_Rγ2-derived eicosapeptide was previously described [19]. To that end, an artificial sequence was created concatenating ACBP/DBI protein sequence with GABA_A_Rγ2-derived eicosapeptide, separated by a linker of 30 glycine residues (N-ACBP/DBI-(Gly)30-GABA_A_Rγ2 peptide). Then, Alphafold (v.2.3.1) was used to predict the resulting molecular structure. The program was run in monomer mode, using all the databases, and modifying the number of recycles from three to nine. The protein structures generated were then analyzed using UCSF ChimeraX visualization software [20]. In order to elucidate the interacting residues in ACBP/DBI- GABA_A_Rγ2 eicosapeptide structure, the H-bonds tool from ChimeraX was run with default settings and the most frequent predicted interactions were annotated.

### Real time interaction analyses

Kinetics of interaction of recombinant murine ACBP/DBI (Institute of Psychiatry and Neuroscience of Paris, France) with GABA_A_Rγ2 -derived eicosapeptide or its mutated derivative (synthesized by Biosynth ltd) was analyzed by surface plasmon resonance-based biosensor system – Biacore 2000 (Cytiva Life Sciences, Biacore, Uppsala, Sweden). SA sensor chip (Cytiva Life Sciences, Biacore) was activated by three consequent injections of 1 M NaCl, 0.05 M NaOH solution. After washing the sensor chip surface, biotinylated ACBP/DBI was immobilized on it. ACBP was diluted in HBS-EP buffer (10 mM HEPES pH 7.2, 150 mM NaCl, 3 mM EDTA, and 0.005% Tween-20) at final concentration of 10 µg/mL and injected for 5 min contact time. The achieved immobilization density of ACBP was 1600 resonance units.

All analyses were performed in HBS-EP buffer (0.01 M HEPES pH 7.4, 0.15 M NaCl, 3 mM EDTA, 0.005% v/v Surfactant P20; 0.22 µm filtered and degassed). The system was run at flow rate of 30 μL/min. The temperature on chip surface was set to 25 °C. GABA_A_Rγ2-derived eicosapeptide or its mutated derivative (both stocked in DMSO at 1 mg/mL) were diluted in HBS-EP to 10 µM and 100 µM. The peptides were injected over immobilized ACBP/DBI and a control surface (streptavidin alone). The association and dissociation phases of the interaction were followed for 4 and 5 min, respectively. The interaction of peptides with ACBP/DBI was assessed by monitoring the dissociation from the immobilized protein. For visualization of the binding response Graph Pad Prism software was used.

### Statistics

The in vivo experiments were performed with 3–9 animals per group. Data were reported as columns or box plots (with each dot representing one biological replicate) including the mean ± SEM. The sample size is noted in the figures. Normality tests and equal variance tests (F or Bartlett) were performed. Statistical significance was analyzed using Mann–Whitney test, unpaired two-tailed Student’s *t*-test, or one-way ANOVA. Differences were considered statistically significant when *p* values were *p* < 0.05 or non-significant (ns) when *p* > 0.05.

## Results

### GABA_A_Rγ2 upregulation by obesogenic diets

**Fig. 1:**
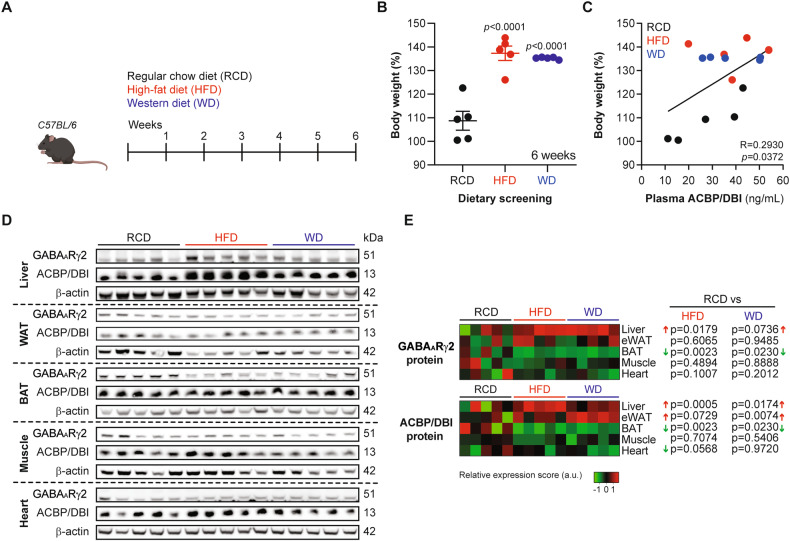
GABA_A_Rγ2 expression across metabolically relevant organs responding to obesogenic diets in mice. **A** Experimental design. C57BL/6 male mice were fed with regular chow diet (RCD), high-fat diet (HFD) or high-fat/high-sucrose/1.25% cholesterol diet (Western diet: WD) over the course of 6 weeks. **B** Body weight gain measurements in RCD-, HFD- or WD-fed mice. Results are displayed as whisker plots with each dot representing one single mouse (*n* = 4–5 mice per condition) including the mean ± SEM. For statistical analysis, *p* values are calculated by one-way ANOVA. **C** Spearman correlation between body weight gain (compared to week 0) and plasma ACBP/DBI concentration in individual mice fed with RCD, HFD or WD (n = 5 mice per group). **D** Liver, epididymal white adipose tissue (eWAT), interscapular brown adipose tissue (BAT), muscle, and heart protein extract immunoblot detection of GABA_A_Rγ2, ACBP/DBI, and β-actin from mice receiving RCD, HFD or WD (*n* = 5 mice per condition). **E** Heatmap representation of ACBP/DBI, and GABA_A_Rγ2 relative protein levels from RCD-, HFD- or WD-fed mice (*n* = 5 mice per condition). Red and green arrows indicate increased and decreased proteins of interest, respectively (compared to RCD). For statistical analysis, *p* values were calculated by one-way ANOVA. a.u. arbitrary units.

**Fig. 2:**
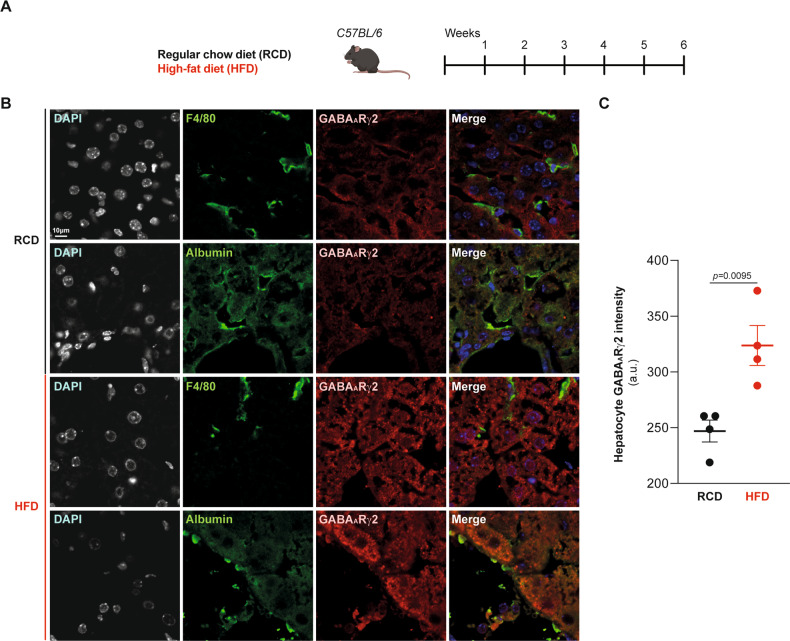
Hepatocyte-specific GABA_A_Rγ2 upregulation in response to obesogenic diets determined by immunofluorescence. **A** Experimental design. C57BL/6 male mice were fed with RCD or HFD over the course of 6 weeks. **B** Representative confocal microscopy images and quantification (**C**) of GABA_A_Rγ2 levels in mice fed with RCD or HFD (*n* = 4 mice per condition). F4/80 and albumin are markers specific for murine macrophages and hepatocytes, respectively. For statistical analyses, *p* values are calculated by two-tailed unpaired Mann–Whitney *U*-test. Scale bar: 10 μm. a.u.: arbitrary units.

**Fig. 3:**
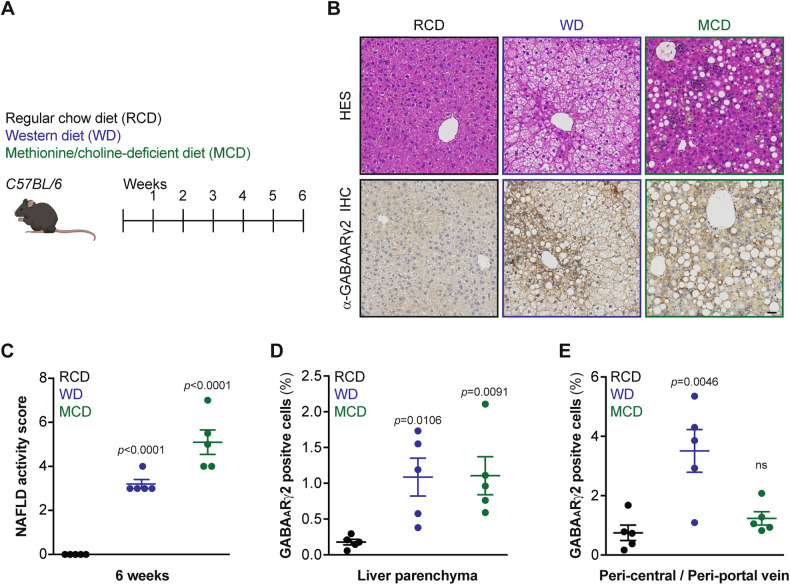
Hepatic GABA_A_Rγ2 upregulation in response to hepatic lipoanabolism determined by immunohistochemistry. **A** Experimental design. C57BL/6 male mice were fed with RCD, WD, and methionine/choline-deficient diet (MCD) over the course of 6 weeks. **B** Representative images of hepatic hematoxylin and eosin (HE), and GABA_A_Rγ2 immunohistochemistry (IHC) staining from mice fed with RCD, WD or MCD (*n* = 5 mice per condition), non-alcoholic fatty liver disease (NAFLD) activity score quantification (**C**). Hepatic GABA_A_Rγ2quantification (expressed as % percentage of GABA_A_Rγ2 -positive cells) in liver parenchyma (**D**) and peri-central / periportal vein (**E**) areas. Results are displayed as whisker plots with each dot representing one single mouse (*n* = 5 mice per condition) including the means ± SEM. For statistical analyses, *p* values are calculated by two-tailed unpaired Mann–Whitney *U*-test (**C**) or one-way ANOVA (**D**, **E**).

C57BL/6 mice were fed with obesogenic diets, either high-fat diet (HFD) or Western style high-fat/sucrose/cholesterol diet (WD) for 6 weeks (Fig. 1A), which yielded similar weight gain (Fig. 1B), correlating with an elevation of plasma ACBP/DBI concentrations (Fig. 1C), in agreement with previous findings [8, 14, 21]. We performed immunoblot analyses using a GABA_A_Rγ2-specific antibody (Fig. S1) in order to determine the levels of the ACBP/DBI receptor in major metabolically relevant organs. We found that GABA_A_Rγ2 increased in the liver, but not in skeletal muscle, heart or epididymal white adipose tissue (WAT) and actually decreased in interscapular brown adipose tissue (BAT) (Fig. 1D, E). Moreover, ACBP/DBI increased in liver and WAT, but remained unaltered in muscle and heart upon exposure to obesogenic diets (Fig. 1D, E). We used two additional methods to corroborate the upregulation of hepatic GABA_A_Rγ2. First, immunofluorescence staining allowed us to colocalize GABA_A_Rγ2 with the hepatocyte-specific marker albumin but not with the macrophage marker F4/80 (Fig. 2) and immunohistochemistry (Fig. 3). Of note, methionine/choline deficient diet (MCD) (which is not obesogenic, but induces hepatosteatosis and inflammation, Fig. 3B, C) also led to a diffuse, plasma membrane-localized upregulation of GABA_A_Rγ2 in the liver parenchyma (Fig. 3B, D), though a less pronounced peri-central/peri-portal upregulation than obesogenic WD (Fig. 3B, E).

Altogether, these results suggest the implication of the ACBP/DBI- GABA_A_Rγ2 system in the liver responding to obesogenic (and to some degree also non-obesogenic, steatogenic) diets.

### Direct interaction between ACBP/DBI and GABA_A_Rγ2-derived eicosapeptide

**Fig. 4:**
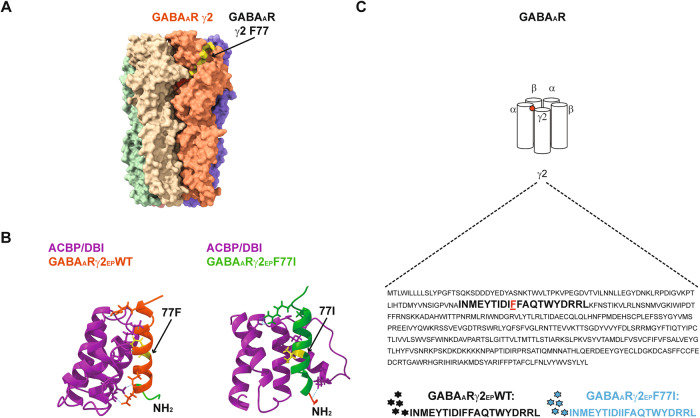
Molecular modeling of the interaction between ACBP/DBI and the GABA_A_Rγ2-derived eicosapeptide. **A** Computational model of GABA_A_R γ2 subunit of GABA_A_R (GABA_A_R γ2) is highlighted in orange color. The binding site of ACBP/DBI on GABA_A_R γ2 (amino acid residues 106–125) is indicated with a black arrow. **B** In silico homology modeling of predicted physical interaction between ACBP/DBI and wild-type (WT) or “phenylalanine 77 to isoleucine”-substitution mutation (F77I) GABA_A_Rγ2 peptides. ACBP/DBI is represented in violet while WT and F77I peptides are represented in orange and green respectively. **C** Schematic representation of amino acid sequence features between WT and F77I GABA_A_Rγ2 peptides (both encoding for the 106–125 amino acid residues of the full GABA_A_Rγ2 protein). WT peptide (black stars) is designed in a way that it is predicted to bind to ACBP while, in contrast, the mutated F77I peptide (blue stars) is designed in a such way that it is predicted that it will not bind to ACBP.

Murine *GABRG2* encodes a part of the heteropentameric GABA_A_R, and large portions of this protein subunit are surface-exposed, as determined by cryogenic electron microscopy and molecular modeling [22]. A point mutant in which the phenylalanine (F) residue in position 77 is replaced by an isoleucine (I) residue (F77I), which is surface exposed as well (Fig. 4A, B), is known to abolish the interaction of GABA_A_R with ACBP/DBI [13, 14]. We derived several 20-mer (eicosa) peptides in which F77 (or as a mutated control F77I) was placed in position 5, 10 or 15 and attempted to synthesize them. Only the eicosapeptides (EP) with F77 (or 77I) in position 10 were obtained at a high abundance and could be conveniently dissolved in dimethylsulfoxide (DMSO) (sequences indicated in Fig. 4C). Of note, the non-modified EP (single letter amino acid code: INMEYTIDIFFAQTWYDRRL that we refer to GABA_A_Rγ2-EP-WT) corresponds to a domain of *GABRG2* that is 100% identical across different vertebrate species including zebrafish, chicken, mouse and human (Fig. S2) but specific for this GABA_A_R gamma chain isoform *GABRB2* from human or mouse, because *GABRG1* bears a ‘natural’ F77I mutation and *GABRG3* bears a threonine-to-glutamine exchange in position 74 (T74Q) (Fig. S3A, B).

Molecular modeling suggested that the wild-type (WT) EP efficiently interacts with ACBP/DBI (Fig. 4B). An asparagine (N) residue in position 75 in GABA_A_Rγ2 has a high probability to form salt bridge interactions with lysine (K) residues in ACBP/DBI (either K13 or K33). Such interactions are likely to be affected by the F77I substitution found in GABA_A_Rγ1, as well as by the T74Q substitution found in GABA_A_Rγ3 (Fig. S3).

**Fig. 5:**
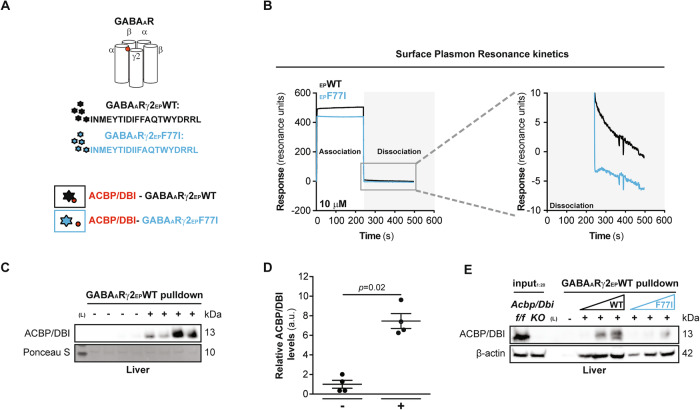
Biophysical and biochemical detection of a direct interaction between ACBP/DBI and the GABA_A_Rγ2-derived eicosapeptide. **A** Schematic representation of the GABA_A_R and the GABA_A_Rγ2-derived eicosapeptides. **B** Surface plasmon resonance kinetics of ACBP-GABA_A_Rγ2-derived eicosapeptide (WT and F77I) interactions. Representative ACBP immunoblot (**C**) from control- or GABA_A_Rγ2-immunoprecipitated samples. Anti-GABA_A_Rγ2 immunoprecipitation (IP) assay was employed in order to validate the GABA_A_Rγ2 WT peptide interaction with native ACBP (in murine hepatic protein extracts), and quantification (**D**). Results are displayed as whisker plots (with each dot representing one single biological replicate) including the mean ± SEM. For statistical analysis *p* value (indicating statistical comparisons with the control condition) was calculated by Mann–Whitney *U*-test. **E** Anti-GABA_A_Rγ2 IP assay to address the physical interaction between GABA_A_Rγ2 peptides and murine hepatic ACBP protein. Interaction was validated using an increasing gradient of biotinylated WT/F77I peptides that were bound on the surface of streptavidin beads as part of the IP assay. Livers from tamoxifen-inducible whole-body knockout mice with floxed *Acbp/Dbi*^*f/f*^ exon 2 (*Acbp/Dbi KO*) and livers from wild-type *Acbp/Dbi*^*f/f*^ control were used as the input controls. kDa kilodaltons, (L): protein ladder. a.u. arbitrary units.

In agreement with the molecular model, GABA_A_Rγ2-EP-WT associated more rapidly with biotinylated recombinant ACBP/DBI immobilized on a surface plasmon resonance chip than did mutated GABA_A_Rγ2-EP-F77I. GABA_A_Rγ2-EP-WT also dissociated more slowly from ACBP/DBI than GABA_A_Rγ2-EP-F77I (Fig. 5A, B). Biotinylated GABA_A_Rγ2-EP-WT immobilized on streptavidin-precoated beads absorbed endogenous ACBP/DBI protein from mouse liver extracts (Fig. 5C, D), and mutated GABA_A_Rγ2-EP-F77I was less efficient in pulling down ACBP/DBI than GABA_A_Rγ2-EP-WT (Fig. 5E).

Altogether, these observations indicate that a linear peptide derived from GABA_A_Rγ2, GABA_A_Rγ2-EP-WT, efficiently interacts with native and recombinant ACBP/DBI. The question then arises whether GABA_A_Rγ2-EP-WT can functionally neutralize ACBP/DBI, thus acting as an ACBP/DBI antagonist.

### Neutralization of ACBP/DBI by GABA_A_Rγ2-derived eicosapeptide

**Fig. 6:**
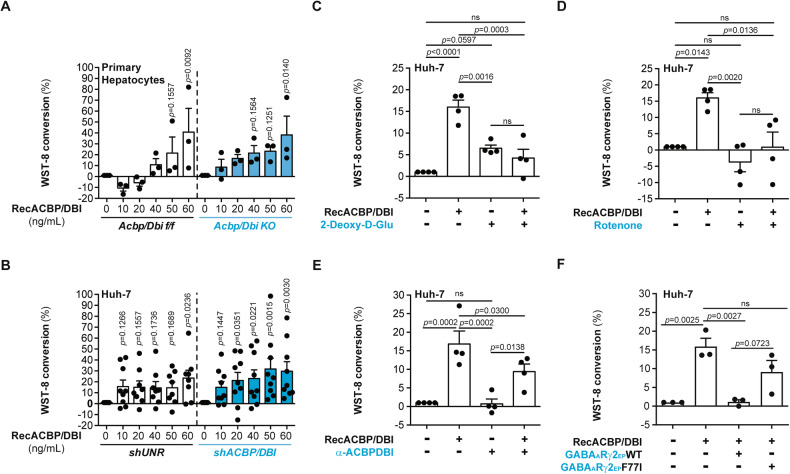
Inhibition of the metabolic effects of ACBP/DBI on hepatocytes by the GABA_A_Rγ2-derived eicosapeptide. **A** WST-8 conversion by murine primary hepatocytes treated with increasing concentrations of recombinant ACBP/DBI protein (RecACBP/DBI) for 4 h. Hepatocytes were isolated from tamoxifen-induced *Acbp/Dbi f/f* or *Acbp/Dbi KO* full-body genetic background mice (white- or blue-colored graphs respectively). Results are displayed as column plots with each dot representing hepatocytes isolated and cultured from one single mouse (*n* = 3 mice per condition) including the means ± SEM. For statistical analysis *p* values are calculated by one-way ANOVA. **B** WST-8 conversion by control (*shUNR*) and *ACBP/DBI*-knocked down (*shACBP/DBI*) human Huh-7 cells treated with increasing concentrations of RecACBP/DBI for 4 h. *shUNR* and *shACBP/DBI* cells are represented by white- or blue-colored graphs respectively. Results are displayed as column plots with each dot representing cells one biological replicate (*n* = 8–9 per condition) including the mean ± SEM. **C** WST-8 conversion by Huh-7 cells treated with RecACBP/DBI or negative control (4 h). Cells were pre-treated with 2-deoxy-D-glucose (2-Deoxy-D-glu; 300 µM) or vehicle (2 h). Results are displayed as column plots with each dot representing cells one biological replicate (*n* = 4 per condition) including the means ± SEM. **D** WST-8 conversion by Huh-7 cells treated with RecACBP/DBI or negative control (4 h). Cells were pre-treated with rotenone (0.5 µM) or vehicle (2 h). Results are displayed as column plots with each dot representing cells one biological replicate (*n* = 4 per condition) including the means ± SEM. **E** WST-8 conversion by Huh-7 cells treated with RecACBP/DBI or negative control (4 h). Prior to administration, RecACBP/DBI was overnight pre-incubated with monoclonal antibody (α-ACBP/DBI) or isotype IgG1. Results are displayed as column plots with each dot representing cells one biological replicate (*n* = 4 per condition) including the means ± SEM. **F** WST-8 conversion by Huh-7 cells treated with RecACBP/DBI or negative control (4 h). Prior to administration, RecACBP/DBI was overnight pre-incubated with GABA_A_Rγ2 (WT or F77I-mutated) eicosapeptides. Results are displayed as column plots with each dot representing cells one biological replicate (*n* = 3 per condition) including the means ± SEM. *p* values are calculated by one-way ANOVA.

Primary mouse hepatocytes or human hepatocellular carcinoma Huh7 cells exposed to increasing concentrations of recombinant ACBP/DBI protein exhibit an increase in the capacity to reduce 2-(2-methoxy-4-nitrophenyl)-3-(4-nitrophenyl)-5-(2,4-disulfophenyl)-2*H* tetrazolium (best known as WST-8) to a yellow-colored formazan dye within 4.5 h. This effect is similarly found for cells subjected to the knockout or knockdown of *ACBP/DBI* (Fig. 6A, B) and is abolished by the glycolysis inhibitor 2-deoxy-D-glucose (Fig. 6C) or the respiratory chain complex I inhibitor rotenone (Fig. 6D), suggesting that it reflects metabolic activation of the cells by ACBP/DBI, in accordance with the known capacity of NAD(P)H to reduce tetrazolium salts [23]. Addition of a monoclonal antibody specific for ACBP/DBI interfered with metabolic activation of Huh7 cells by ACBP/DBI (Fig. 6E). Similarly, GABA_A_Rγ2-EP-WT (but not GABRG2-EP-F77I) inhibited the ACBP/DBI-stimulated metabolic activation of Huh7 cells (Fig. 6F). We also took advantage of transgenic mice expressing microtubule-associated proteins 1A/1B light chain 3B (hereafter referred to as LC3) fused to green fluorescent protein (GFP). We obtained purified hepatocytes from such GFP-LC3-expressing transgenic mice, which were cultured in the presence of leupeptin (to block the final steps of autophagic flux) alone or in combination with the GABA_A_Rγ2-EP-WT for 16 h. We observed that the GABA_A_Rγ2-EP-WT increased the number of LC3-GFP dots observable by means of a confocal microscope (Supplementary Fig. 4). These results plead in favor of the capacity of the GABA_A_Rγ2-EP-WT to enhance autophagic flux.

**Fig. 7:**
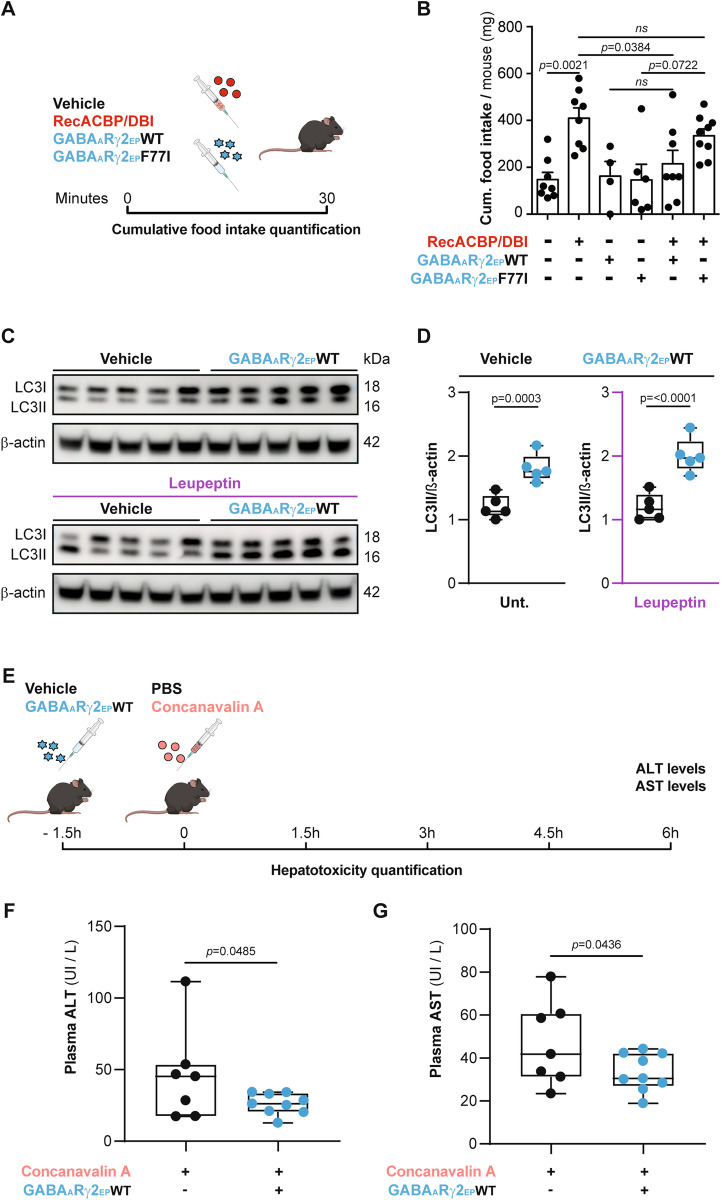
In vivo inhibition of ACBP/DBI function by the GABA_A_Rγ2-derived eicosapeptide. **A**, **B** Appetite stimulation by ACBP/DBI. RecACBP/DBI alone or combined with GABA_A_Rγ2-derived eicosapeptides (EP) (WT or F77I) was administered in vivo by intravenous (i.v.) injection and individual cumulative food intake was measured after 30 min. GABA_A_Rγ2 peptides are represented as blue stars, while murine RecACBP/DBI is represented as red circles (**A**). Results (**B**) are displayed as column plots with each dot representing one single mouse (n = 4 to 9 mice per condition) including the means ± SEM. *p* values were calculated by two-way ANOVA. **C**, **D** Immunoblots and densitometric quantification of LC3 lipidation in liver tissues from mice treated with GABA_A_Rγ2-derived eicosapeptides (EP) WT with or without the autophagy flux inhibitor leupeptin (*n* = 5 mice per group). *p* values were calculated by unpaired one-tailed Student’s *t*-test. **E**–**G** Inhibition of concanavalin A hepatotoxicity. GABA_A_Rγ2 WT peptide (or negative vehicle) was i.v. injected to C57BL/6 male mice followed by concanavalin A (or negative vehicle) administration 1.5 h later (**E**). Alanine transaminase (ALT) (**F**) and aspartate transaminase (AST) (**G**) activity in plasma was analyzed by means of a colorimetric assay (*n* = 7–9 mice per condition). *p* values were calculated by unpaired one-tailed Student’s *t*-test. ns: non-significant. Unt: Untreated.

In the next step, we investigated the capacity of GABA_A_Rγ2-EP-WT to interfere with the function of ACBP/DBI in vivo. For this, we first intravenously injected C57BL/6 mice with recombinant ACBP/DBI protein alone or in combination with GABA_A_Rγ2-EP-WT (or its mutated control GABA_A_Rγ2-EP-F77I) and measured food intake 30 min after administration (Fig. 7A). As previously reported [8], recombinant ACBP/DBI induced an increase in appetite. This hyperphagy effect of ACBP/DBI was abolished by GABA_A_Rγ2-EP-WT but not by its mutated counterpart GABA_A_Rγ2-EP-F77I (Fig. 7B). In the next experiment, we investigated whether GABA_A_Rγ2-EP-WT might inhibit endogenous ACBP/DBI as well. Indeed, we have previously described that antibody-mediated ACBP/DBI neutralization induces autophagy in the liver [8] and reduces the acute hepatotoxic effect of the lectin concanavalin A [9]. Moreover, we have observed that activation of autophagy in the liver was induced by the injection of GABA_A_Rγ2-EP-WT peptides (Fig. 7C, D). Preconditioning the mice with GABA_A_Rγ2-EP-WT shortly before concanavalin A injection significantly blunted the surge in plasma levels of alanine and aspartate aminotransferases (ALT and AST), two enzymes that are released from damaged and dying hepatocytes (Fig. 7E–G) [9].

In sum, these data suggest that GABA_A_Rγ2-EP-WT might act as an ACBP/DBI antagonist and hence block the effects of exogenous recombinant ACBP/DBI in vitro and in vivo, as it also interferes with the effect of endogenous (hepatotoxicity-enabling) ACBP/DBI in vivo.

## Discussion

When in excess, the interaction between extracellular ACBP/DBI and the γ2 subunit of GABA_A_R can be highly pathogenic, contributing to cell loss, inflammation and fibrosis (as discussed in the “Introduction”) in the liver. This also applies to other organs. Thus, the ACBP/DBI neutralization protects against lung fibrosis induced by bleomycin, as well as against myocardium infarction [9] and anthracycline-induced cardiac aging [24, 25]. Moreover, ACBP/DBI knockout protects against stroke in a mouse model [26]. It is known that several of these conditions responding to ACBP/DBI neutralization are accompanied by elevations of circulating ACBP/DBI plasma levels, as documented for obesity [8, 10], non-alcoholic steatohepatitis [12] and systemic inflammation [27]. In addition, as shown here, it appears that liver damage induced by obesogenic nutrition as well as non-obesogenic (but hepatosteatosis-inducing) methionine/choline deficient diet is accompanied by the upregulation of the ACBP/DBI receptor GABA_A_Rγ2 on hepatocytes. This further supports the potential pathogenic effects of ACBP/DBI on metabolically active cells.

The appetite stimulatory and metabolic disease-inducing effects of ACBP/DBI are lost in mice bearing a point mutation (F77I) in *Gabrg2* that abolishes the receptor-ligand interaction [9, 16]. This finding pleads in favor of the idea that GABA_A_R receptors containing this particular γ2 subunit (*GABRG2* instead of *GABRG1* or *GABRG3*) are the sole responders to extracellular ACBP/DBI. Accordingly, the F77I mutation occurs ‘naturally’ in *GABRG1*, contrasting with *GABRG3* that encodes for an F residue in this position but has exchanged another amino acid (T74Q) only 4 positions upstream. In contrast, F77I and the stretch of amino acids flanking F77I in *GABRG2* are 100% conserved across vertebrate evolution. An eicosapeptide containing 9 amino acids *N*-terminal from F77 and 10 amino acids C-terminal from F77 (i.e., the GABA_A_Rγ2-EP-WT peptide with the sequence INMEYTIDIFFAQTWYDRRL) was able to interact with ACBP/DBI in silico, in biophysical assays involving recombinant ACBP/DBI protein, as well as in pulldown experiments performed on liver extracts containing native ACBP/DBI. These reactivities were attenuated for a mutated eicosapeptide carrying the F77I mutation. Functional experiments confirmed that GABA_A_Rγ2-EP-WT efficiently blocked the effects of recombinant human ACBP/DBI protein on human hepatocytes, as well as the hyperphagy-stimulatory effects of mouse ACBP/DBI protein in vivo, commensurate with its capacity to attenuate acute hepatotoxicity induced by concanavalin A. Thus, GABA_A_Rγ2-EP-WT showed similar effects as antibodies specific for ACBP/DBI in thus far that it acts as an ACBP/DBI antagonist.

The aforementioned results may pave the way to the development of peptidomimetics or – more generally – small molecules blocking the interaction between ACBP/DBI and GABA_A_Rγ2 or – more specifically – the binding of ACBP/DBI to GABA_A_Rγ2-EP-WT. This latter interaction can be measured by surface plasmon resonance, greatly facilitating high-throughput screens. Such screens could be preceded by artificial intelligence-assisted preselection of molecules, the structure of which likely interferes with the binding of ACBP/DBI and GABA_A_Rγ2 in silico, as predicted by molecular modeling. That said, after more than a decade of pharmacological and clinical development, antibodies that block the – in economic terms – most lucrative protein-protein interaction between PD-1 and PD-L1 have not been replaced by small molecules (to date there has only been one successful clinical trial [28]), illustrating the difficulty to achieve the substitution of antibodies by small molecule entities. Notwithstanding this caveat, it will be important to pursue the development of synthetic, orally available ACBP/DBI antagonists for therapeutic interventions on metabolic disease.

### Supplementary information

Supplementary materials

WB Raw datas

## Data Availability

The datasets generated during and/or analysed during the current study are available from the corresponding author on reasonable request.
