# Supplementary material for: Inhibition of acyl-CoA binding protein (ACBP) by means of a GABAARγ2-derived peptide
Source: Cell Death Dis. 2024 Apr 6;15(4):249. doi: 10.1038/s41419-024-06633-6 (PMC10998878; doi:10.1038/s41419-024-06633-6)
Supplement: Supplementary file 1 — Supplementary materials [file 41419_2024_6633_MOESM1_ESM.pdf]

Inhibition of Acyl-CoA Binding Protein (ACBP) by Means of a GABA$_A$Rγ2-Derived Peptide

Gerasimos Anagnostopoulos[1,2*], Ester Saavedra[1,2,3*], Flavia Lambertucci[1,2], Omar Motiño[1,2], Jordan Dimitrov[4], David Roiz-Valle[5], Victor Quesada[5,8], Karla Alvarez-Valadez[1,2,6] Hui Chen[1,2,6], Allan Sauvat[1,2], Yan Rong[1,2,6], Uxía Nogueira-Recalde[1,2,7], Sijing Li[1,2,6], Léa Montégut[1,2], Mojgan Djavaheri-Mergny[1,2], Maria Castedo[1,2], Carlos Lopez-Otin[1,5,9], Maria Chiara Maiuri[1,2,10], Isabelle Martins[#1,2] and Guido Kroemer[#1,2,11]

Affiliations

[1] Centre de Recherche des Cordeliers, Equipe labellisée par la Ligue contre le cancer, Université de Paris, Sorbonne Université, Inserm U1138, Institut Universitaire de France, Paris, France
[2] Metabolomics and Cell Biology Platforms, Institut Gustave Roussy, Villejuif, France
[3] Departamento de Bioquímica y Biología Molecular, Fisiología, Genética e Inmunología, Instituto Universitario de Investigaciones Biomédicas y Sanitarias (IUIBS), Universidad de Las Palmas de Gran Canaria, Las Palmas de Gran Canaria, Spain
[4] Centre de Recherche des Cordeliers, INSERM, CNRS, Sorbonne Université, Université Paris Cité, Paris, France
[5] Departamento de Bioquímica y Biología Molecular, Instituto Universitario de Oncología (IUOPA), Universidad de Oviedo, Oviedo, Spain
[6] Faculté de Médecine, Université de Paris Saclay, Kremlin Bicêtre, France
[7] Grupo de Investigación en Reumatología (GIR). Instituto de Investigación Biomédica de A Coruña (INIBIC), Fundación Profesor Novoa Santos, A Coruña, Spain
[8] Centro de Investigación Biomédica en Red de Cáncer (CIBERONC), Madrid, Spain
[9] Facultad de Ciencias de la Vida y la Naturaleza, Universidad Nebrija, Madrid, Spain.
[10] Department of Molecular Medicine and Medical Biotechnologies, University of Napoli Federico II, 80131 Naples, Italy.
[11] Institut du Cancer Paris CARPEM, Department of Biology, Hôpital Européen Georges Pompidou, AP-HP, Paris, France

* Gerasimos Anagnostopoulos and Ester Saavedra contributed equally to this work

# Correspondence: Guido Kroemer (kroemer@orange.fr) or Isabelle Martins (isabelle.martins@inserm.fr)

Supplemental Figures

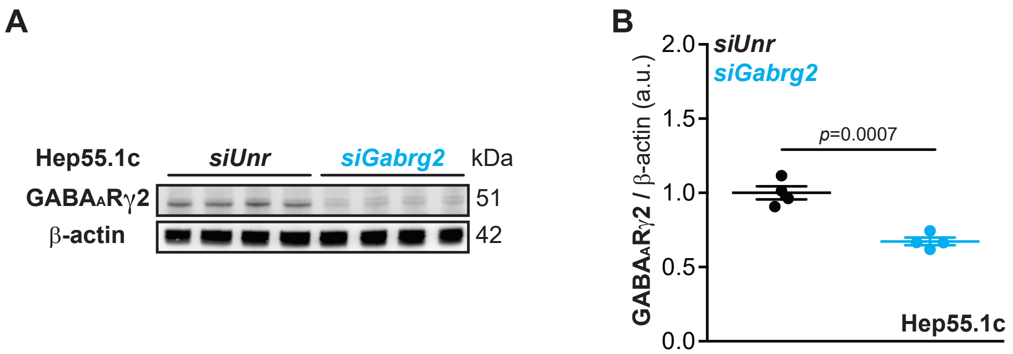
A
Hep55.1c
siUnr
siGabrg2
kDa
GABAARγ2
51
β-actin
42
B
2.0
1.5
1.0
0.5
0.0
siUnr
siGabrg2
p=0.0007
GABAARγ2 / β-actin (a.u.)
Hep55.1c

Figure S1

Figure S1.

(A) Representative immunoblot images of murine $GABA_AR\gamma2$ and β-actin proteins in control (*siUnr*) and *Gabrg2*-silenced (*siGabrg2*) Hep55.1c cells, densitometric quantification **(B).** For statistical analysis *p* values were calculated by unpaired two-tailed Student's t-test. kDa: kilodaltons. a.u.: arbitrary units.

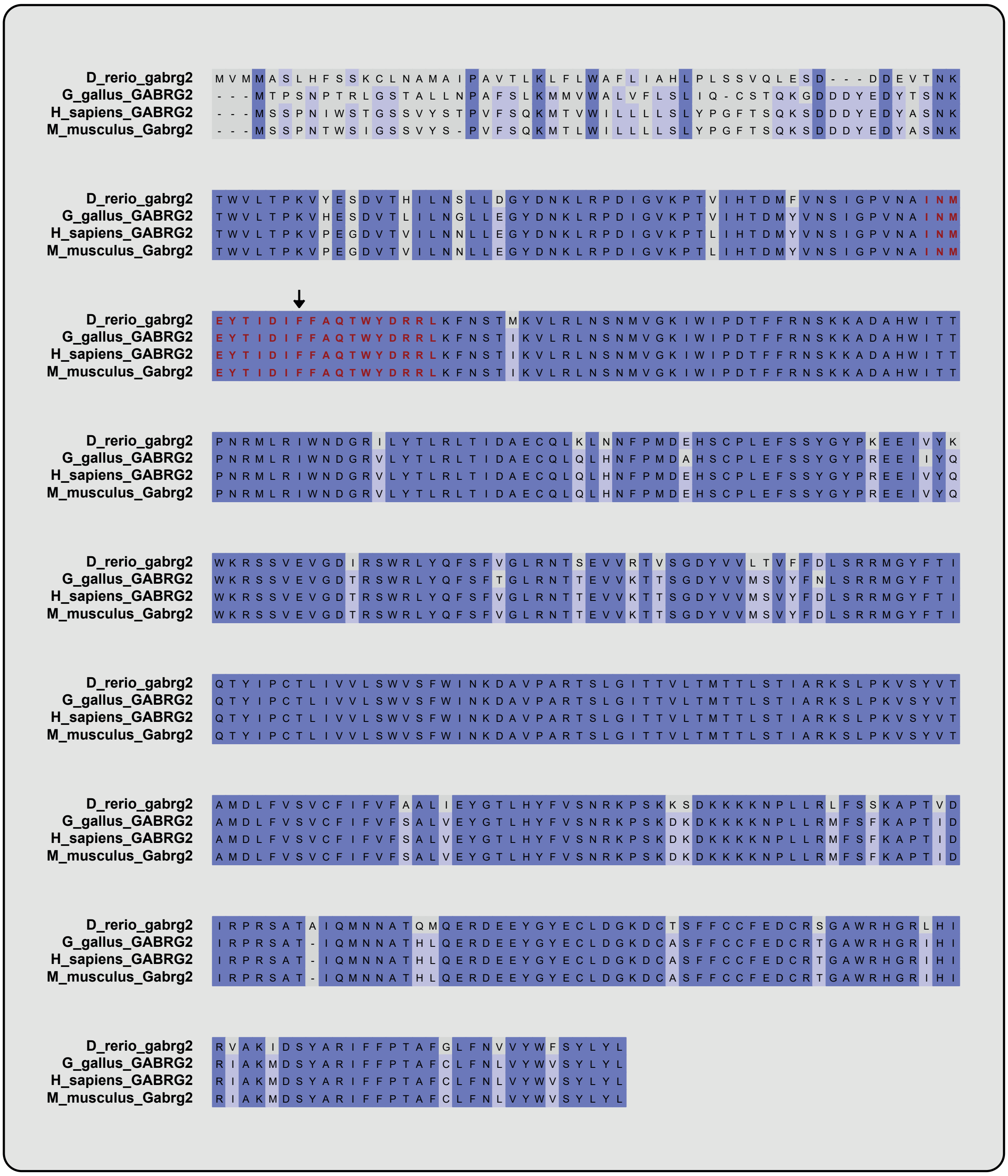
D_rerio_gabrg2 MVMMASLHFSSKCLNAMAIPAVTLKLFLWAFLIAHLPLSSVQLESD---DDEVTNK
G_gallus_GABRG2 ---MTPSNPTRLGSTALLNPAFSLKMMVWALVFLSLIQ-CSTQKGDDDYEDYTSNK
H_sapiens_GABRG2 ---MSSPNIWSTGSSVYSTPVFSQKMTVWILLLLSLYPGFTSQKSDDDYEDYASNK
M_musculus_Gabrg2 ---MSSPNTWSIGSSVYS-PVFSQKMTLWILLLLSLYPGFTSQKSDDDYEDYASNK
D_rerio_gabrg2 TWVLTPKVYESDVTHILNSLLDGYDNKLRPDIGVKPTVIHTDMFVNSIGPVNAINM
G_gallus_GABRG2 TWVLTPKVHESDVTLILNGLLEGYDNKLRPDIGVKPTVIHTDMYVNSIGPVNAINM
H_sapiens_GABRG2 TWVLTPKVPEGDVTVILNNLLEGYDNKLRPDIGVKPTLIHTDMYVNSIGPVNAINM
M_musculus_Gabrg2 TWVLTPKVPEGDVTVILNNLLEGYDNKLRPDIGVKPTLIHTDMYVNSIGPVNAINM
D_rerio_gabrg2 EYTIDIFFAQTWYDRRLKFNSTMKVLRLNSNMVGKIWIPDTFFRNSKKADAHWITT
G_gallus_GABRG2 EYTIDIFFAQTWYDRRLKFNSTIKVLRLNSNMVGKIWIPDTFFRNSKKADAHWITT
H_sapiens_GABRG2 EYTIDIFFAQTWYDRRLKFNSTIKVLRLNSNMVGKIWIPDTFFRNSKKADAHWITT
M_musculus_Gabrg2 EYTIDIFFAQTWYDRRLKFNSTIKVLRLNSNMVGKIWIPDTFFRNSKKADAHWITT
D_rerio_gabrg2 PNRMLRIWNDGRILYTLRLTIDAECQLKLNNFPMDEHSCPLEFSSYGYPKEEIVYK
G_gallus_GABRG2 PNRMLRIWNDGRVLYTLRLTIDAECQLQLHNFPMDAHSCPLEFSSYGYPREEIIYQ
H_sapiens_GABRG2 PNRMLRIWNDGRVLYTLRLTIDAECQLQLHNFPMDEHSCPLEFSSYGYPREEIVYQ
M_musculus_Gabrg2 PNRMLRIWNDGRVLYTLRLTIDAECQLQLHNFPMDEHSCPLEFSSYGYPREEIVYQ
D_rerio_gabrg2 WKRSSVEVGDIRSWRLYQFSFVGLRNTSEVVRTVSGDYVVLTVFFDLSRRMGYFTI
G_gallus_GABRG2 WKRSSVEVGDTRSWRLYQFSFTGLRNTTEVVKTTSGDYVVMSVYFNLSRRMGYFTI
H_sapiens_GABRG2 WKRSSVEVGDTRSWRLYQFSFVGLRNTTEVVKTTSGDYVVMSVYFDLSRRMGYFTI
M_musculus_Gabrg2 WKRSSVEVGDTRSWRLYQFSFVGLRNTTEVVKTTSGDYVVMSVYFDLSRRMGYFTI
D_rerio_gabrg2 QTYIPCTLIVVLSWVSFWINKDAVPARTSLGITTVLTMTTLSTIARKSLPKVSYVT
G_gallus_GABRG2 QTYIPCTLIVVLSWVSFWINKDAVPARTSLGITTVLTMTTLSTIARKSLPKVSYVT
H_sapiens_GABRG2 QTYIPCTLIVVLSWVSFWINKDAVPARTSLGITTVLTMTTLSTIARKSLPKVSYVT
M_musculus_Gabrg2 QTYIPCTLIVVLSWVSFWINKDAVPARTSLGITTVLTMTTLSTIARKSLPKVSYVT
D_rerio_gabrg2 AMDLFVSVCFIFVFAALIEYGTLHYFVSNRKPSKKSDKKKKNPLLRLFSSKAPTVD
G_gallus_GABRG2 AMDLFVSVCFIFVFSALVEYGTLHYFVSNRKPSKDKDKKKKNPLLRMFSFKAPTID
H_sapiens_GABRG2 AMDLFVSVCFIFVFSALVEYGTLHYFVSNRKPSKDKDKKKKNPLLRMFSFKAPTID
M_musculus_Gabrg2 AMDLFVSVCFIFVFSALVEYGTLHYFVSNRKPSKDKDKKKKNPLLRMFSFKAPTID
D_rerio_gabrg2 IRPRSATAIQMNNATQMQERDEEYGYECLDGKDCTSFFCCFEDCRSGAWRHGRLHI
G_gallus_GABRG2 IRPRSAT-IQMNNATHLQERDEEYGYECLDGKDCASFFCCFEDCRTGAWRHGRIHI
H_sapiens_GABRG2 IRPRSAT-IQMNNATHLQERDEEYGYECLDGKDCASFFCCFEDCRTGAWRHGRIHI
M_musculus_Gabrg2 IRPRSAT-IQMNNATHLQERDEEYGYECLDGKDCASFFCCFEDCRTGAWRHGRIHI
D_rerio_gabrg2 RVAKIDSYARIFFPTAFGLFNVVYWFSYLYL
G_gallus_GABRG2 RIAKMDSYARIFFPTAFCLFNLVYWVSYLYL
H_sapiens_GABRG2 RIAKMDSYARIFFPTAFCLFNLVYWVSYLYL
M_musculus_Gabrg2 RIAKMDSYARIFFPTAFCLFNLVYWVSYLYL

Figure S2

Figure S2.

Amino-acid residue sequence alignment of GABRG2 proteins from *Danio rerio*, *Gallus gallus*, *Homo sapiens*, and *Mus musculus*. The red-font residues correspond to the exact sequence of the $GABA_AR\gamma2$ peptides that were designed and used in this study (identical in all four species). The black arrow highlights the phenylalanine (F) 77 (in the mutated background of which the ACBP/DBI – $GABA_AR\gamma2$ interaction is abolished). The blue shading suggests partial or absolute conservation among GABRG isoforms.

Note that the amino acid numbering is based on the assumption that the first residue would be the methionine encoded by the start codon in the immature GABRG2 proteins, explaining the position of "F77", which has been numerated based on a nomenclature in which the first residues is found in the proteolytically processed, mature GABRG2 protein.

The alignments were performed using the tool https://degradome.uniovi.es/cgi-bin/vqf/alig.cgi.

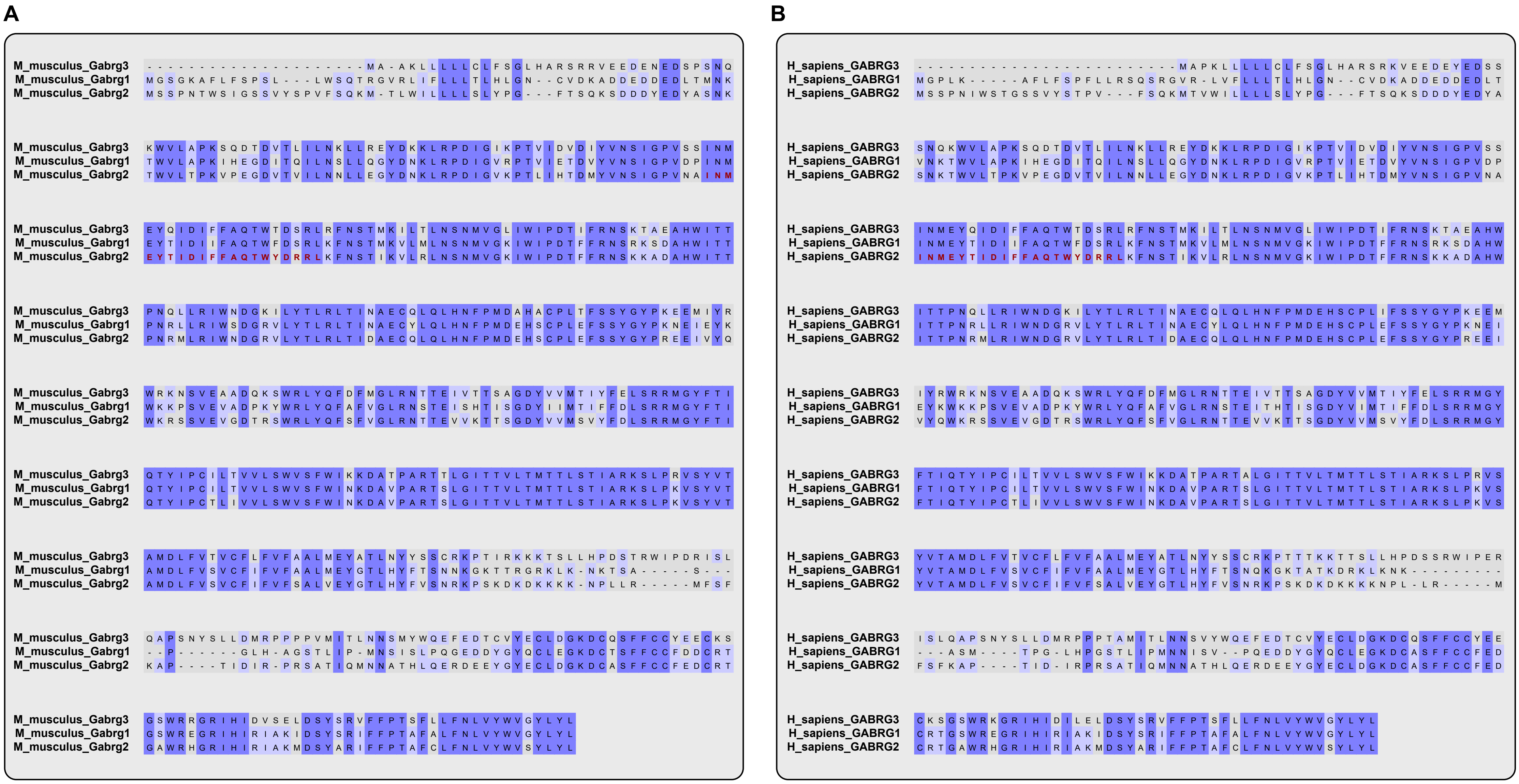
A
M_musculus_Gabrg3
M_musculus_Gabrg1
M_musculus_Gabrg2
B
H_sapiens_GABRG3
H_sapiens_GABRG1
H_sapiens_GABRG2

Figure S3

Figure S3.

Amino-acid residue sequence alignment GABRG1, GABRG2, GABRG3 proteins from *Mus musculus* (**A**) and *Homo sapiens* (**B**). The red-font residues correspond to the sequence of the $GABA_A$Rγ2 peptides that were designed and used in this study (only conserved in GABRG2). The blue shading suggests partial or absolute conservation among GABRG isoforms.

The alignments were performed using the tool https://degradome.uniovi.es/cgi-bin/vqf/alig.cgi.

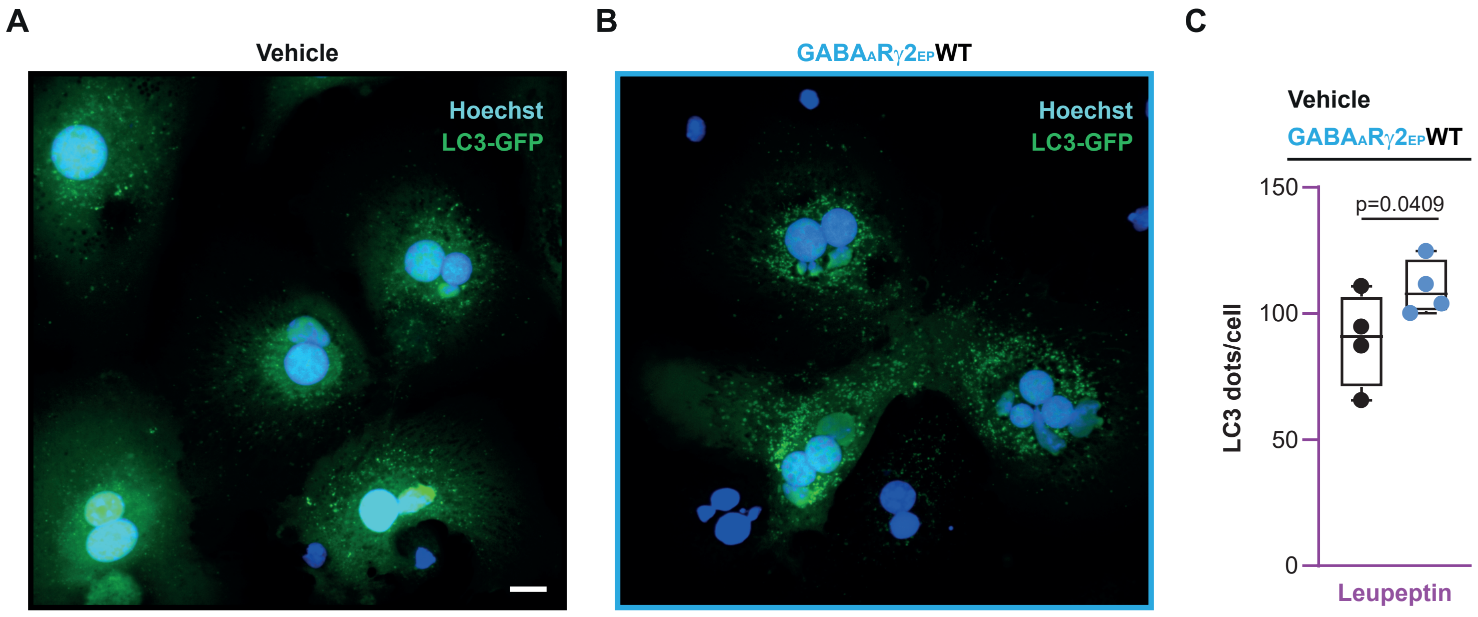

A
Vehicle
Hoechst
LC3-GFP
B
GABAARγ2EPWT
Hoechst
LC3-GFP
C
Vehicle
GABAARγ2EPWT
p=0.0409
LC3 dots/cell
150
100
50
0
Leupeptin

Figure S4

Figure S4.

Hepatocytes were isolated from 4 transgenic mice expressing microtubule-associated proteins 1A/1B light chain 3B (hereafter referred to as LC3) fused to green fluorescent protein (GFP) (resulting fusion protein: LC3-GFP) under a ubiquitous promoter. Such oligo- or monocellular hepatocyte suspensions were cultured in the continuous presence of leupeptin at 37°C in the absence (**A**) or presence (**B**) of the GABA$_A$Rγ2-EP-WT (INMEYTIDIFFAQTWYDRRL) for 18 hours. Cells were counterstained with Hoechst 33342 and representative fluorescence images are shown (bar size: 10 µm). The number of LC3-GFP+ dots per cells (defined as an area of interest around the 1 or 2 Hoechst 33342-positive nuclei per cell) were determined by image analysis and plotted in **C**. Results are shown as means ± standards of the mean, and statistics were calculated with a paired Student t test.
