# Supplementary material for: Inhibition of acyl-CoA binding protein (ACBP) by means of a GABAARγ2-derived peptide
Source: Cell Death Dis. 2024 Apr 6;15(4):249. doi: 10.1038/s41419-024-06633-6 (PMC10998878; doi:10.1038/s41419-024-06633-6)

Figure 1

BAT
Line up

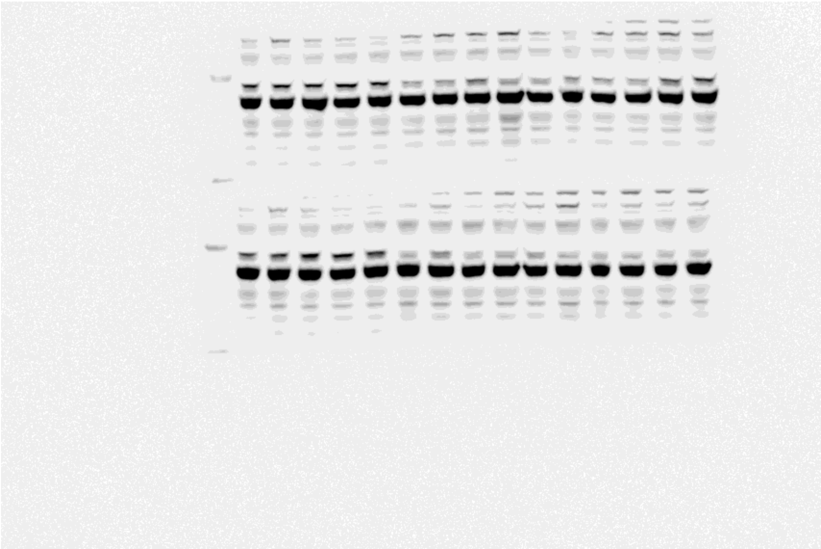

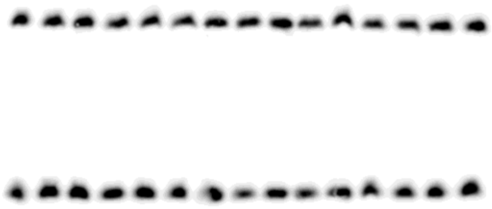

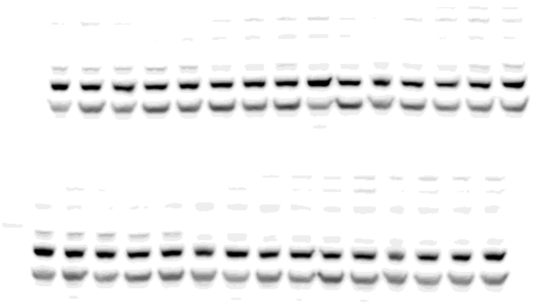

Figure 1

Heart

Line up

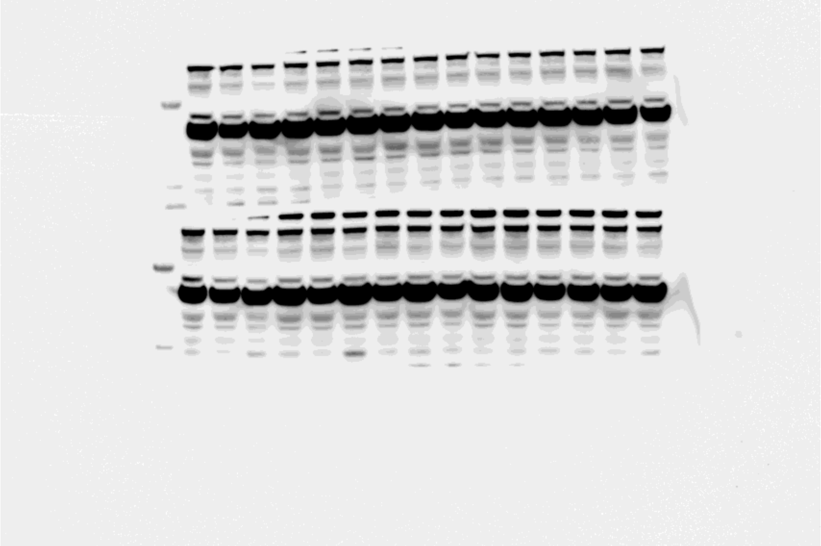

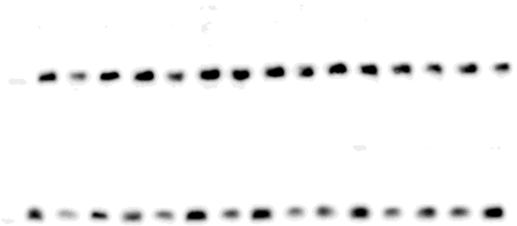

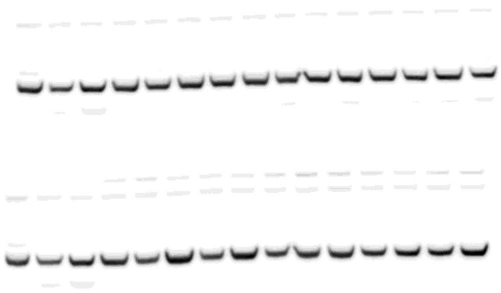

Figure 1

Liver
Line up

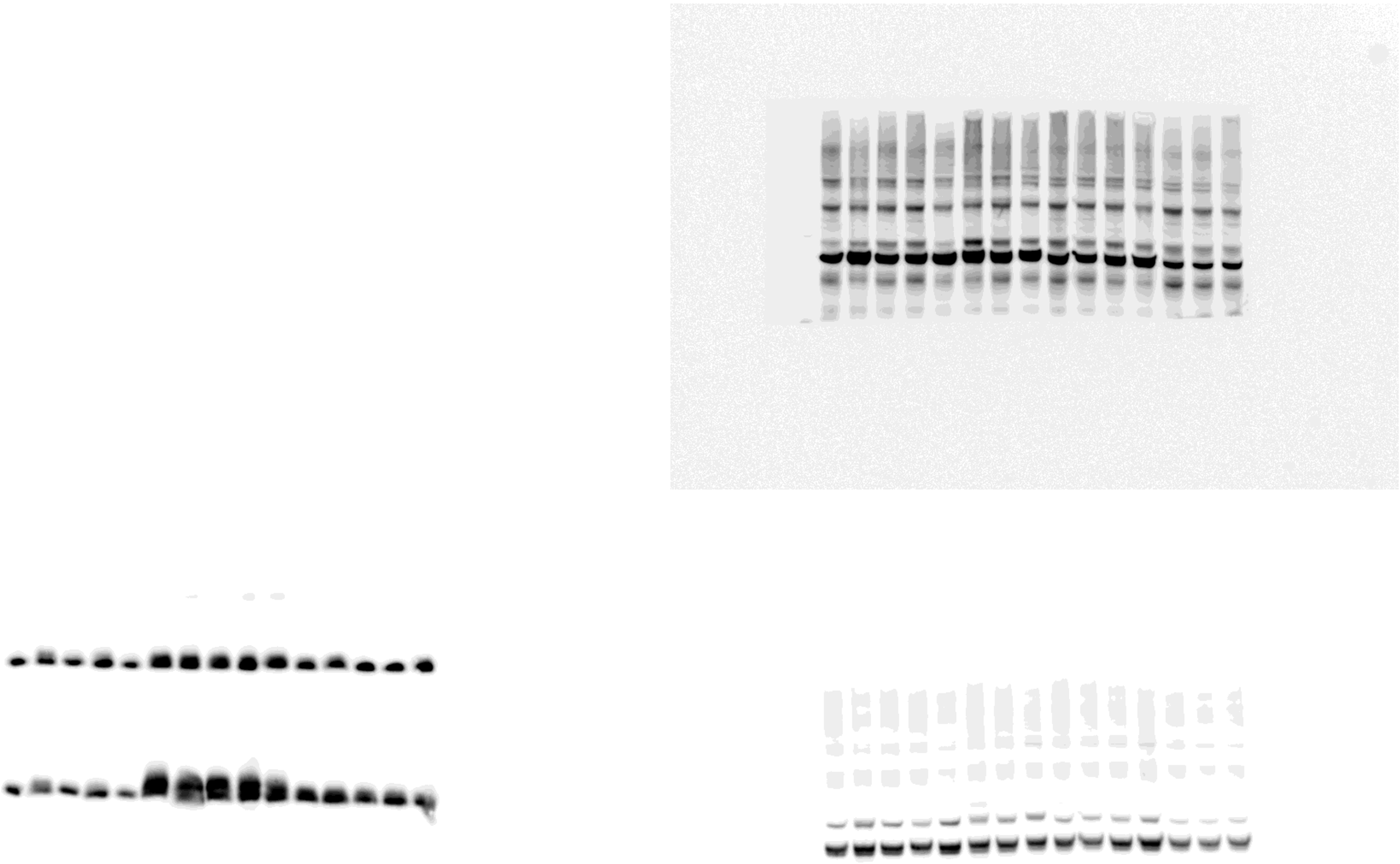

Figure 1

Muscle
Line up

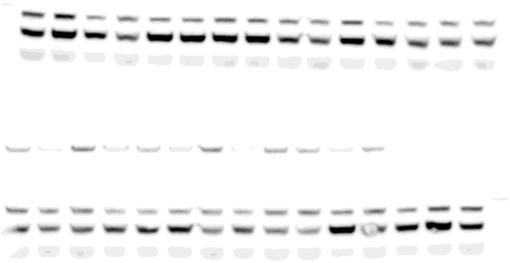

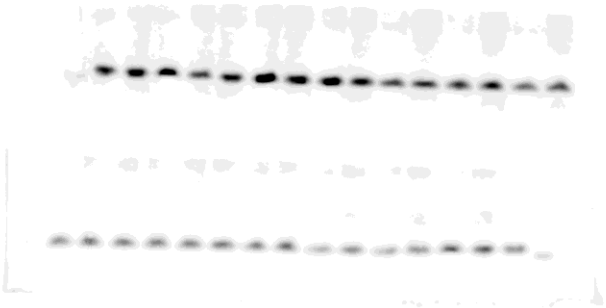

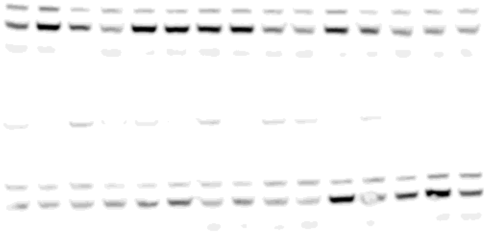

Figure 1

WAT
Line down

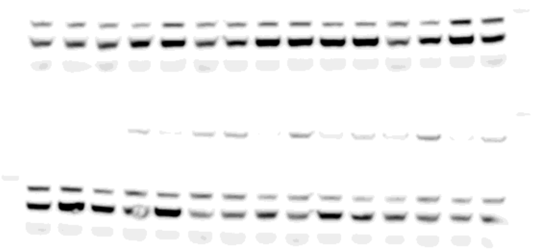

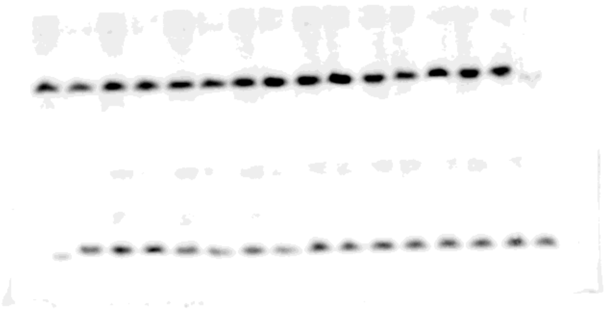

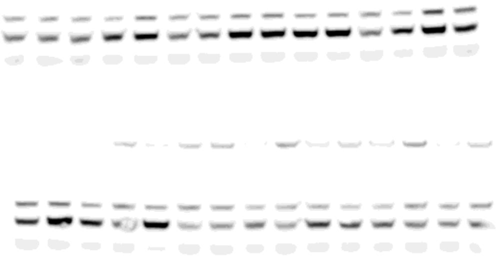

Figure 5

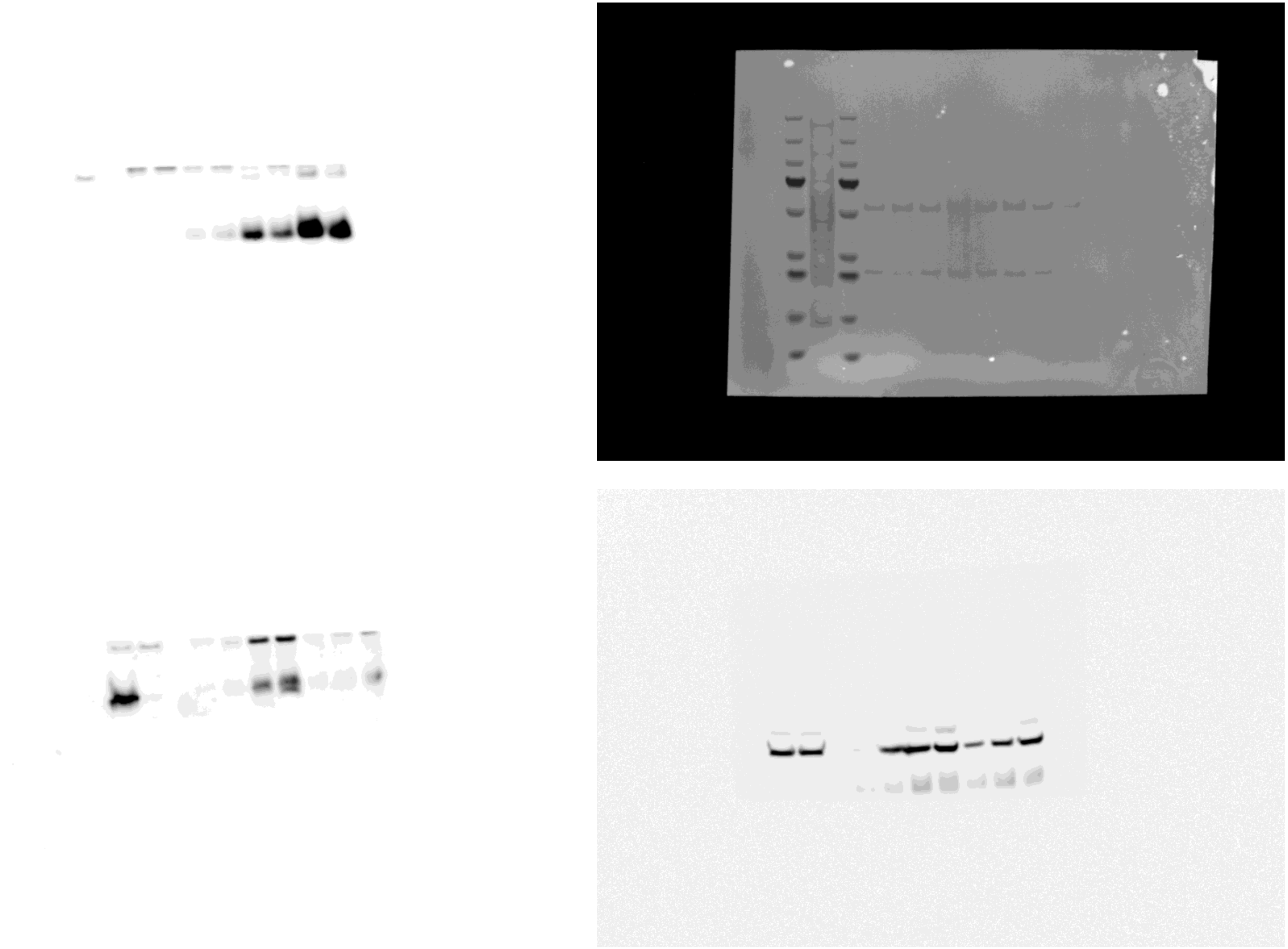

Figure 7

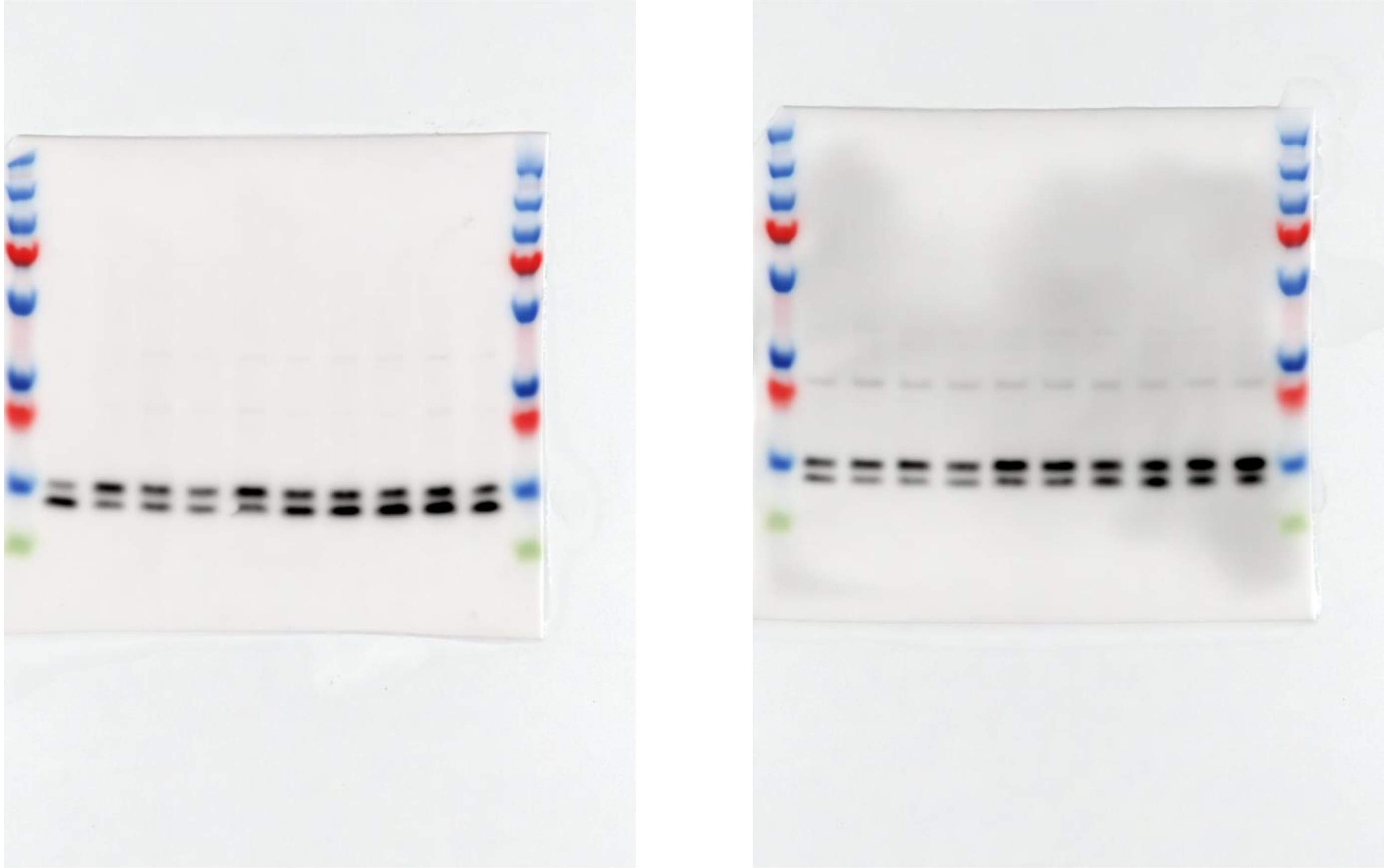

Figure 7

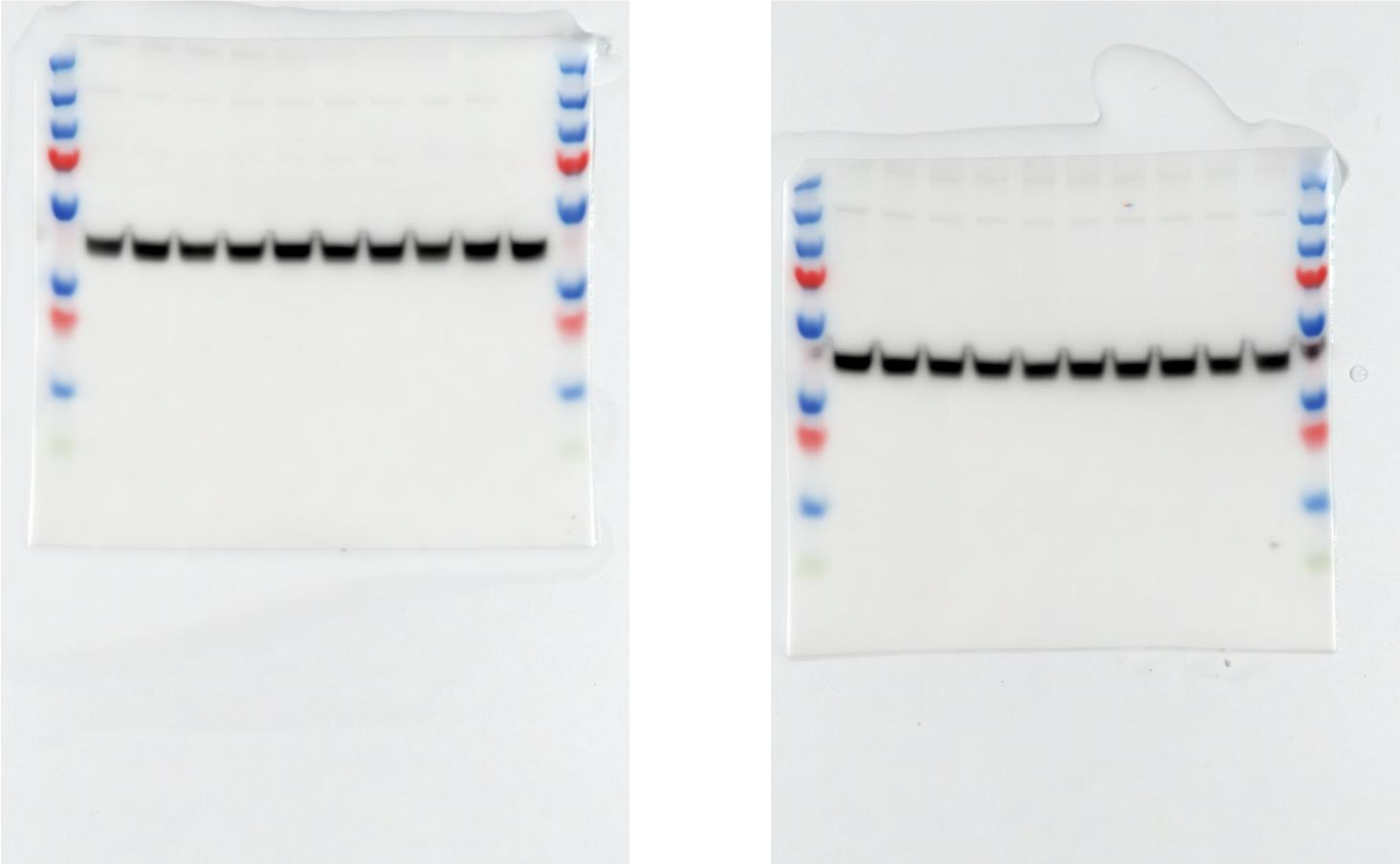

Figure S1

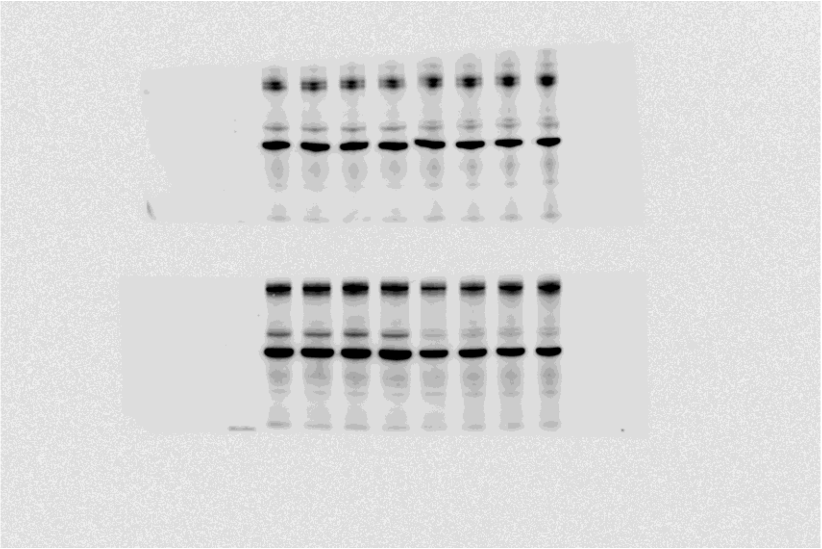

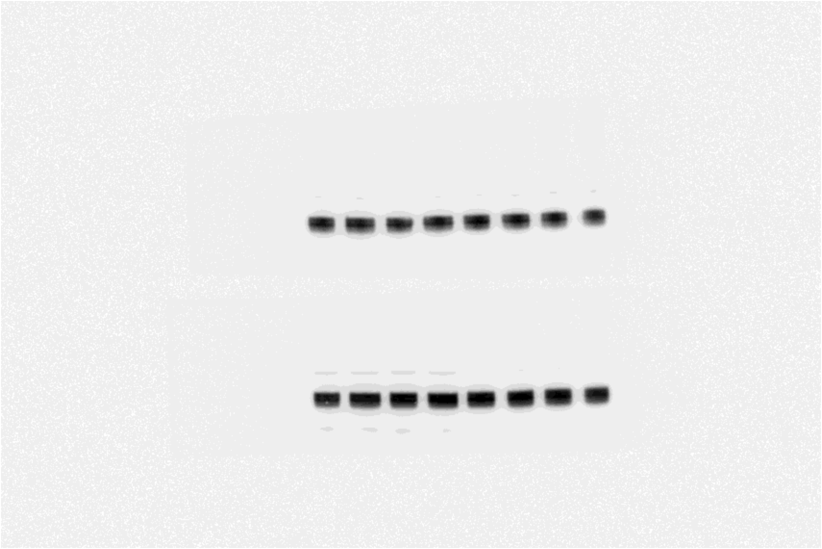

Supplement: Supplementary file 2 — WB Raw datas [file 41419_2024_6633_MOESM2_ESM.pdf]
